# Barbecue conditions affect contents of oxygenated and non-oxygenated polycyclic aromatic hydrocarbons in meat and non-meat patties

**DOI:** 10.1016/j.fochx.2022.100351

**Published:** 2022-05-27

**Authors:** Lisa Zastrow, Michael Judas, Karl Speer, Karl-Heinz Schwind, Wolfgang Jira

**Affiliations:** a Department of Safety and Quality of Meat, Max Rubner-Institut (MRI), E.-C.-Baumann-Straße 20, 95326 Kulmbach, Germany; b Chair of Special Food Chemistry and Food Production, Technical University Dresden, Bergstraße 66, 01069 Dresden, Germany

**Keywords:** OPAH, PAH, Grill, Beef, Meat substitutes, Correlations

## Abstract

The contents of eight oxygenated polycyclic aromatic hydrocarbons (OPAHs; anthracene-9,10-dione, benzo[*a*]anthracene-7,12-dione, 11*H*-benzo[*b*]fluorene-11-one, 6H-benzo[*cd*]pyren-6-one, 7H-benzo[*de*]anthracene-7-one, 9,10-dihydro-8H-benzo[*a*]pyren-7-one, fluoren-9-one, and naphthacene-5,12-dione) and six PAHs (anthracene, fluorene, and PAH4) were investigated in barbecued meat and non-meat patties. The patties were prepared with ten setups (six replicates, each) of barbecue conditions defined by grill type, grate height, heating medium, and barbecue time. The highest median contents were observed with a disposable grill (OPAHs: 46.3 µg/kg; PAHs: 40.7 µg/kg) and a charcoal grill (OPAHs: 29.6 µg/kg; PAHs: 23.3 µg/kg). Fluoren-9-one and anthracene-9,10-dione were the dominant compounds within OPAHs, but also the four toxicologically most relevant OPAHs were detected with a total up to 11.8 µg/kg. Pairs of OPAHs and corresponding PAHs did not show strong correlations, as individual OPAHs and PAHs were affected differently by the barbecue conditions. No suitable markers for OPAH prediction could be found. We recommend to include OPAHs in future PAH investigations.

## Introduction

Oxygenated polycyclic aromatic hydrocarbons (OPAHs) are a group of derivatives of polycyclic aromatic hydrocarbons (PAHs) with at least one carbonyl or hydroxyl group attached to the aromatic ring structure (Lundstedt et al., 2007). Similar to the PAHs, they are generated during the incomplete combustion of organic material, but also by secondary oxidation of PAHs. Due to their functional group(s), they have a higher polarity, are more persistent, and can spread further in the environment than non-oxygenated PAHs (Lundstedt et al., 2007, Walgraeve et al., 2010, Yu, 2002). OPAHs with carbonyl groups (quinones and ketones) are considered “dead-end products” of many biological and chemical degradation processes (Lundstedt et al., 2007).

For a long time, OPAHs have been neglected in terms of risk assessment. However, an increasing number of studies show that OPAHs, especially quinones and ketones, are suspected to be of significant toxicological relevance to human health, although the mechanism of toxicity has not been completely understood (Lundstedt et al., 2007). Due to their ability to undergo enzymatic or non-enzymatic redox cycles, forming reactive oxygen species, the quinones possess higher cell toxicity than the PAHs (Bolton et al., 2000, Niu et al., 2017). Furthermore, OPAHs can directly attack the DNA and other macromolecules resulting in a direct mutagenic and genotoxic potential, whereas the PAHs first require enzymatic activation in metabolism (Bolton et al., 2000, Lundstedt et al., 2007, Walgraeve et al., 2010, Yu, 2002). Some studies found that OPAHs are more toxic to aquatic organisms than the non-oxygenated PAHs (Brack et al., 2003, Lampi et al., 2006). In addition, OPAHs have been related to lung cancer (Ho et al., 2016). Anthracene-9,10-dione (ATQ), formerly used as a pesticide, is the only OPAH listed with EU maximum residue levels of 10–20 µg/kg for products of plant or animal origin (EU, 2014). However, there exist no legal regulations regarding process related OPAH contamination in food as there are for PAHs (EU, 2011).

Although OPAHs have been included in various analytical environmental studies for several years (Clergé, Le Goff, Lopez, Ledauphin & Delépée, 2019), they are usually not considered in foodstuff assessment. To date, only a small number of foodstuffs or preparation methods known to generate PAHs have also been checked for OPAHs. The majority of papers related to OPAHs in foodstuffs studied their presence in vegetable oils (Gong et al., 2018a, Hua et al., 2016, Teng et al., 2019, Zhao et al., 2018) or products prepared with them, e.g., fried peanuts (Zhao, Wu, Gong, Li & Zhuang, 2017) or fried bread (Gong et al., 2018b, Li et al., 2016). Three of these studies investigated the influence of oil refining or different storage conditions (Gong et al., 2018a, Hua et al., 2016, Zhao et al., 2018). Here, five OPAHs (ATQ, benzo[*a*]anthracene-7,12-dione (BaAQ), 7H-benzo[*de*]anthracene-7-one (BZA), 9,10-dihydro-8H-benzo[*a*]pyren-7-one (BaPO), and fluoren-9-one (9FLO)) were analyzed, and a total content up to 23 µg/kg was detected. Even higher OPAH contents up to 40 µg/kg were found by Teng et al. (2019) in waste cooking oil. Various OPAHs have also been detected in coffee, tea infusions, milk, or milk powders (Anggraini et al., 2020, dos Santos et al., 2019, Yan et al., 2021). Chen et al. (2014) found the highest levels with about 80 µg/kg for four selected OPAHs (ATQ, BaAQ, BZA, and 9FLO) in meat smoked directly over fireplaces for several weeks. ATQ has also been detected in industrially smoked sausages (Zastrow, Schwind, Schwägele & Speer, 2019). In all these studies, the OPAHs were detected in the same order of magnitude as the corresponding PAHs.

Barbecuing has a long tradition and is still very popular. However, especially barbecuing with charcoal is known to be a source of PAH contamination (Iko Afé et al., 2020). In order to design strategies to reduce the PAH content in barbecued products, some studies on the influence of different conditions before, during and after barbecuing have already been carried out (Iko Afé et al., 2020). Well-known factors influencing the PAH content are the type of fuel, the duration of barbecuing, the fat content of the product, the use of marinades, or the preparation of the charcoal (Chaemsai et al., 2016, Gorji et al., 2016, Iko Afé et al., 2020, Lee et al., 2016, Oz and Yuzer, 2016). For example, products prepared on an electric grill have lower PAH contents than those prepared on a charcoal grill (Iko Afé et al., 2020), products with low fat content also have lower PAH contents (Gorji et al., 2016, Lee et al., 2016), and the PAH content can be reduced by preheating the charcoal before barbecuing (Chaemsai et al., 2016).

To the best of our knowledge, there have been no studies on the occurrence of OPAHs in barbecued products or on the influence of different barbecue conditions. Previously, we developed a method for the simultaneous analysis of PAHs and selected relevant OPAHs (ATQ, BaAQ, BaPO, 11*H*-benzo[*b*]fluorene-11-one (BbFLO), 6H-benzo[*cd*]pyren-6-one (BcdPO), BZA, naphthacene-5,12-dione (NAPHQ), and 9FLO) and showed that barbecuing with a charcoal grill can produce considerable amounts of these OPAHs (Zastrow, Speer, Schwind & Jira, 2021). Based on these results, the aim of the present study was to investigate if the OPAH content can be reduced or affected by different barbecue conditions. Also, correlations between OPAHs and PAHs should be investigated, and we wanted to clarify if an unknown OPAH content can be predicted from a measured PAH content. For this purpose, three different patty types (beef, vegetarian, and vegan) were barbecued with various barbecue setups. In addition to grill type, also heating medium and grate height were varied. This approach should adequately reflect the range of barbecue setups as used by consumers.

## Materials and methods

*Reagents and materials*

All solvents were of picograde or optigrade quality. Acetone, cyclohexane, ethyl acetate (EA), and acetonitrile (ACN) were obtained from LGC Standards (Wesel, Germany). *N*-dodecane (anhydrous, ≥ 99%), poly(acrylic acid), partial sodium salt-*graft*-poly(ethylene oxide), cross-linked, 90–850 μm, and the Supelclean tubes EZ-POP NP (2.5 g, 12 mL) were purchased from Sigma Aldrich (Munich, Germany). The native OPAHs ATQ, BaAQ, BaPO, BbFLO, BcdPO, BZA, NAPHQ, and 9FLO were purchased from Chiron AS (Trondheim, Norway). Fluorene (FLU) was obtained from Sigma Aldrich (Munich, Germany). A standard mixture of the native PAH4 (benzo[*a*]anthracene (BaA), benzo[*a*]pyrene (BaP), benzo[*b*]fluoranthene (BbF), and chrysene (CHR)), a standard solution of anthracene (ANT), and the deuterated ANT-d_10_ were purchased from Restek (Bad Homburg, Germany). The deuterated compounds ATQ-d_8_ and FLU-d_10_ were obtained from Chiron AS (Trondheim, Norway). The standard mixture of the deuterated PAH4 (BaA-d_12_, CHR-d_12_, BbF-d_12_, and BaP-d_12_) was prepared by mixing the single compounds purchased from CDN Isotopes (Augsburg, Germany). The isotope-labeled compounds CHR-^13^C_6_ and fluoranthene-^13^C_6_ obtained from LGC Standards (Wesel, Germany) were used as injection standards. For the barbecue setups, a charcoal grill ‘Venezuela’ and disposable grills from Activia Mastercook (HM Heiße Metallwaren, Hof, Germany), a contact grill ‘Silex S-162′ from Silex (Hamburg, Germany), an electric grill ‘Q 140′ and a gas grill ‘Q 220′ from Weber (Ingelheim, Germany), and an indirect charcoal grill ‘Lotusgrill classic’ from LotusGrill (Limburgerhof, Germany) were used. The charcoal and the charcoal briquettes were from proFagus (Bodenfeld, Germany), and the barbecue lighting cubes from Boomex (Essen, Germany).

*Barbecue samples*

In ten experimental setups that were defined by combinations of grill type, heating medium, and grate height, barbecue samples were prepared from commercial beef patties (Table 1). We assumed that a grate height of 2 cm is standard in practical application, and we used a charcoal grill with either lump charcoal or charcoal briquettes, and in addition a disposable grill with this grate height. The effect of an excessively long barbecue time was tested with the charcoal briquette grill only, by barbecuing the patties until they had burnt areas, regardless of core temperature (Briq2e in Table 1). For these four setups, barbecue samples from non-meat patties, namely vegetarian patties based on milk protein, and vegan patties based on wheat protein, were also prepared. The detailed composition of the three patty types is given in Supplementary Table S1. In addition, samples from beef patties only were prepared with four grill types that are known for a relatively low contamination with PAHs: indirect, gas, contact, and electric grill. Also, with beef patties only, the potential reduction in PAHs and OPAHs by increasing the grate height for the charcoal briquette grill to 4 or 8 cm was analyzed (Table 1).

**Table 1:** Process parameters applied in ten experimental setups with one or three patty types (with six replicates each, total *n* = 108).

**Label**^A^	**Grill type**	**Heating medium**	**Grate Height** **(cm)**^C^	**Temperature** **(°C)**^D^	**Preheating time**	**Barbecue time (min)**^F^		
					**(min)**^E^	**Beef**^G^	**Vegan**^G^	**Vegetarian**^G^
Briq2	Charcoal grill	Charcoal briquettes 1200 g	2	229	15	7	6	6
Briq4	Charcoal grill	Charcoal briquettes 1200 g	4	254	15	7	–	–
Briq8	Charcoal grill	Charcoal briquettes 1200 g	8	185	15	8	–	–
Briq2e B	Charcoal grill	Charcoal briquettes 1200 g	2	240	15	14	9	9
Disp2	Disposable grill	Charcoal briquettes 700 g	2	179	25	18	15	15
Char2	Charcoal grill	Lump charcoal 900 g	2	211	20	7	6	6
Ind	Indirect grill	Lump charcoal 160 g	–	186	15	20	–	–
Cont0	Contact grill	Electric	0	200	2	2	–	–
Elec4	Electric grill	Electric	4	148	20	18	–	–
Gas7	Gas grill	Butane gas	7	221	10	8	–	–

A Briq: briquettes; Disp: disposable; Ind: indirect; Cont: contact; Elec: electric. The digit represents the grate height.

B Extended barbecue time.

C Distance between the grill grate and the heating medium.

D Mean temperature at the level of the grill grate.

E Time between ignition of the heating medium and starting of the barbecue time.

F The barbecue time was set after preliminary tests, based on the time required to reach a defined core temperature of the patties, separately for beef or non-meat patties. The patties were turned over once after half the barbecue time.

G Weights and sizes of the patties (weight/diameter/thickness): beef 100 g/10 cm/1 cm; vegan 100 g/10 cm/1.2 cm; vegetarian 80 g/9 cm/1.2 cm.

The 18 combinations of barbecue setup and patty type were carried out with six replicates each (in total *n* = 108; Table 1). Each replicate was composed of three individual patties, and two replicates of each combination were prepared consecutively on one of three different days. The patties were frozen for storage at −18 °C and thawed overnight at 6 °C before barbecuing. For each patty type, the barbecue time necessary for reaching a core temperature of 75 °C was determined during preliminary tests. The patties were turned over once after half the barbecue time. During the barbecue process, the temperature at the level of the grill grate was recorded. With all grills, except for the contact grill, the trials were performed outdoors in a covered and wind-protected location to keep the environmental influences as low as possible. With the disposable charcoal and the non-charcoal grills, the grill was prepared and preheated anew for each replicate. For all other charcoal grills, the two replicates of each day were barbecued consecutively with the same lump charcoal or briquettes (Table 1).

With all charcoal grills, except for the disposable one, the lump charcoal or charcoal briquettes were weighed into a lighting chimney and ignited with five pieces of barbecue lighter. After the preheating time, the completely glowing material was transferred to the grill and the grill grate was installed. The contact grill was preheated to 200 °C, and for this setup only, the patties were wrapped in aluminum foil. For the disposable grills, the charcoal briquettes were ignited with the enclosed wax paper. The electric and the gas grills were preheated with lid closed and the highest temperature setting (see Table 1). Then, the lid was kept open during the whole barbecue time. The fire box of the indirect grill (see Supplementary Figure S1) was loaded with lump charcoal and inserted into the grill. To ignite the charcoal, about 6 g of lighting paste was used, and the integrated fan was set to 50% intensity. After preheating, the fan was set to 100% intensity.

The three patties of each replicate were homogenized together as one sample using a Grindomix GM 200 (Retsch, Haan, Germany). All samples were stored in sterile side-seal vacuum bags at –18 °C.

*Analysis of OPAHs and PAHs*

The barbecued patties were analyzed for eight OPAHs und six PAHs (see Supplementary Table S2). The exact procedure and validation of the analytical method used here were described by Zastrow et al. (2021). The samples were prepared in three steps: (i) preparation of the extraction cell with homogenized sample, drying agent and internal standard; (ii) accelerated solvent extraction using an ACN/EA mixture (1/3, v/v); (iii) solid phase extraction with an ACN/EA mixture (97/3, v/v).

Gas chromatography was performed with a Trace Ultra gas chromatograph (Thermo Fisher Scientific, Dreieich, Germany). The injector was operated in the splitless mode at 280 °C. The chromatographic separation was performed with an Rxi®-PAH column (60 m × 0.25 mm × 0.10 μm) from Restek (Bad Homburg, Germany). The injection volume was 1.5 µL, and helium was used as carrier gas with a constant flow of 1 mL/min. The following temperature program was applied: isothermal at 50 °C for 0.1 min, at 30 °C/min to 175 °C, at 6 °C/min to 265 °C, at 4 °C/min to 290 °C, at 30 °C/min to 320 °C and isothermal at 320 °C for 10 min. The high-resolution mass spectrometry analysis with a magnetic sector mass spectrometer DFS (Thermo Fisher Scientific, Dreieich, Germany) was performed in the electron impact positive ion mode. The electron energy was 40 eV, and the temperatures of the transfer line and the ion source were 270 °C and 260 °C, respectively. The resolution of the mass spectrometer was set to 8,000 (10% valley definition). Limit of detection (LOD) and limit of quantification (LOQ) of this analytical method are given in Supplementary Table S2.

### Statistical analysis

The statistical analysis and the calculations were performed with JMP 15 (SAS Institute Inc., Heidelberg, Germany) and Excel (Microsoft Office 2019). The results are based on six replicates. Since not all data were normally distributed, non-parametric tests as well as medians were used for evaluation. Boxplots show median, upper and lower quartiles, and whiskers are a maximum of 1.5 times the interquartile distance, with outliers displayed as dots. The data were tested for significant differences with the Wilcoxon test at p < 0.05. Quadratic regression models of the entire, unweighted dataset were constructed to assess the correlations between OPAHs and PAHs. The coefficients of the linear (X) and quadratic (X^2^) term, the intercept, and the adjusted coefficient of determination (R^2^_adj_) were calculated. If the quadratic term was not significant, linear regression was used. If the intercept was insignificant in linear or quadratic regression, a regression-without-intercept was used. To estimate the prediction quality, the absolute and relative root mean square errors (RMSE) were calculated. In a principal component analysis (PCA) of the entire, unweighted dataset, a biplot was constructed for the first two principal components (for computation and interpretation of PCA, see Kohler & Luniak (2005)).

## Results and discussion

### OPAH and PAH contents in barbecue samples

The patties were barbecued in ten experimental setups which differed in barbecue parameters that can affect the formation and content of OPAHs and PAHs (e.g., grill type or grate height; Table1). The beef patties were prepared within each of the ten setups, while both the vegan and vegetarian meat substitute patties were prepared with the four most relevant setups only. In at least one of all experimental setups, seven of the eight OPAHs and all six PAHs could be detected and quantified (see Supplementary Figure S2). In contrast to meat smoked by Chen et al. (2014), BaPO was not detected in any of the samples analyzed here. For BaP and PAH4, no sample exceeded the respective maximum levels for barbecued meat or meat products of 5 and 30 µg/kg set by Regulation (EU) No. 835/2011.

With contact grill, electric grill, or gas grill, OPAH and PAH contents were the lowest overall. Here, ATQ was the only substance detected, but only as low as 0.3 µg/kg. All other OPAHs and PAHs were below LOD or LOQ. Several studies already reported that the use of electric or gas grills caused the lowest concentrations of PAHs in barbecued meat (Duedahl-Olesen et al., 2015, Rose et al., 2015). However, the patties prepared in the present study also had lower contents of PAH4 than gas-grilled beef kebab (3.2–3.8 µg/kg) and satay samples (3.1–43.7 µg/kg) (Gorji et al., 2016, Nor Hasyimah et al., 2020).

**Fig. 1:**
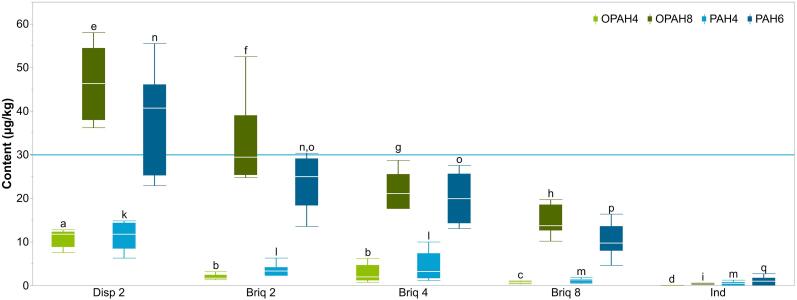
Contents of OPAH4, OPAH8, PAH4, and PAH6 in beef patties barbecued with three grill types and different distances between heating medium and grill grate. For detailed barbecue parameters, see Table 1. The reference line at 30 µg/kg indicates the maximum level for PAH4 in barbecued meat according to Regulation (EU) No. 835/2011. (OPAH4: sum of BaAQ, BbFLO, BcdPO, and NAPHQ; OPAH8: sum of OPAH4, ATQ, BaPO, BZA, and 9FLO; PAH4: sum of BaA, BaP, BbF, and CHR; PAH6: sum of PAH4, ANT, and FLU; *n* = 6, each; different letters represent significant differences between experimental setups within OPAH or PAH group (Wilcoxon test, p = 0.05)).

Of the different grill setups with lump charcoal or charcoal briquettes, the disposable grill (Disp2) produced the overall highest contents of OPAHs and PAHs in beef patties, and the indirect grill (Ind) the lowest (Fig. 1). Chen et al. (2014) analyzed four OPAHs in smoked meat and detected total levels up to 80 µg/kg. The samples barbecued in this study, which were analyzed for eight OPAHs, were all considerably below this concentration.

For the charcoal grill with briquettes, the effect of the grill height, increasing from Briq2 to Briq4 to Briq8, was tested. The barbecue time for grate height 8 cm was extended from 7 to 8 min to compensate the reduction of the average temperature at the grill grate from 230 to 250 °C down to 185 °C (Table1). With increasing distance, median OPAH8 decreased from 29.4 to 13.7 µg/kg, and PAH6 from 24.9 to 9.7 µg/kg. OPAH4 and PAH4 did not differ between Briq2 and Briq4 but decreased for Briq8 from around 1.9 to 0.6 µg/kg, and from about 3.3 to 1.3 µg/kg, respectively. This corresponds to a decrease of OPAH4 by 62%, OPAH8 by 53%, PAH4 by 63%, and PAH6 by 61%. The PAH4 content of Briq8 was comparable to the results of a previous study where the PAH4 content of beef patties, also barbecued for 8 min on a charcoal grill with a distance of 8 cm, was 1.6 µg/kg (García-Lomillo, Viegas, Gonzalez-SanJose & Ferreira, 2017).

The disposable grill Disp2 caused the highest median contents of 46.3 µg/kg OPAH8, and 40.7 µg/kg PAH6 (Fig. 1). Compared to the charcoal grill Briq2 with the same 2 cm grill height, both OPAH8 and PAH6 increased by about 60%. This increase was even more evident for the high-molecular-weight OPAH4 and PAH4, whose contents increased by about 600% and 250%, respectively, to median 11.8 µg/kg, each. Even the ATQ content of this grill type (median: 15.7 µg/kg) exceeded the EU maximum level for pesticide residues in animal tissue of 10 µg/kg (EU, 2014). Disposable grills are sold commercially as ready-to-use sets with a limited amount of charcoal briquettes. Due to their design, they cannot be refilled. In the tests performed here, the limited amount of charcoal briquettes resulted in lower temperatures at the grill grate compared to the charcoal grill. Consequently, the barbecue time had to be doubled to reach the desired core temperature of 75 °C (Table 1). This longer exposure of the patties probably resulted in the higher contents. An increase in PAH4 content from < LOQ to 0.9 µg/kg by increasing the barbecue time from 6 min to 10.5 min was observed by Oz et al. (2016) in barbecued beef steaks. To keep the PAH content as low as possible, it is important that the charcoal is completely smoldering before barbecuing (Chaemsai et al., 2016, Iko Afé et al., 2020). Due to bad ventilation during fire-starting and the resulting low oxygen supply, a complete smoldering of the briquettes and complete combustion of the wax paper (which was included in the set to be used as a barbecue lighter) were not possible with the disposable grills used in this study. Also, the temperatures during the industrial production of lump charcoal and charcoal briquettes have an influence on the PAH content during barbecuing (Chaemsai et al., 2016, Kim et al., 2021). However, the production temperatures of the two types of charcoal briquettes used for Briq2 or Disp2 were unknown to us.

The indirect charcoal grill (Ind) produced considerably lower levels of OPAHs and PAHs (Fig. 1). The contents of OPAH8 and PAH6 were as low as 2 and 4% compared to Briq2, respectively. Within the group of OPAHs, only ATQ could be detected above the LOQ and resulted in 0.4 µg/kg. The six PAHs were determined at low levels of 0.1–0.3 µg/kg, each. These results can be explained by the indirect construction of this grill type, which prevents fat and meat juices from dripping into the heat source (see Supplementary Figure S1). In addition, the built-in fan promotes a better oxygen supply and, consequently, a more complete combustion than conventional charcoal grills, and thus can dissipate smoke to prevent interaction with the barbecued meat (Lee et al., 2016).

**Fig. 2:**
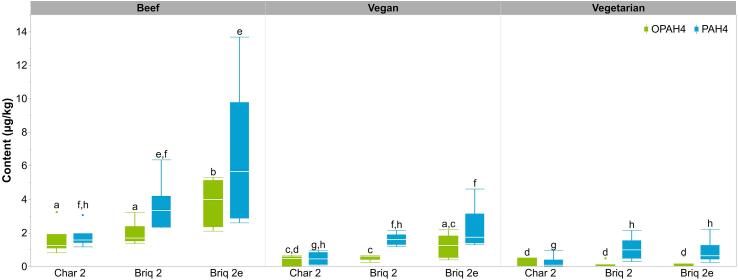
Contents of OPAH4 and PAH4 in various charcoal grill setups, separated by patty type. For detailed barbecue parameters, see Table 1. (OPAH4: sum of BaAQ, BbFLO, BcdPO, and NAPHQ; PAH4: sum of BaA, BaP, BbF, and CHR; vegan: wheat protein based; vegetarian: milk protein based; *n* = 6, each; different letters represent significant differences between experimental setups within OPAH4 or PAH4 (Wilcoxon test, p = 0.05)).

**Fig. 3:**
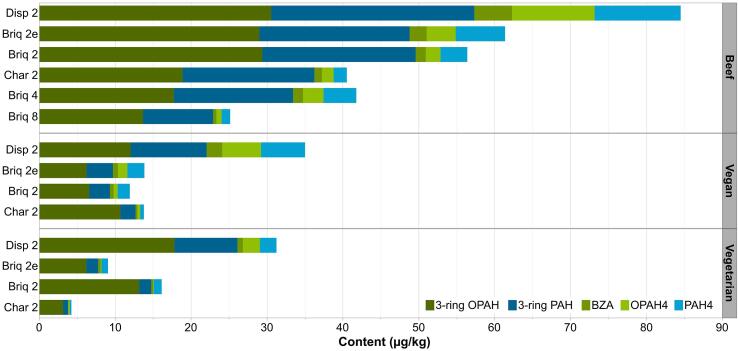
Contents of 3-ring OPAHs, 3-ring PAHs, BZA, OPAH4, and PAH4 in various experimental setups separated by patty type. For detailed barbecue parameters, see Table 1. (3-ring OPAH: sum of ATQ and 9FLO; 3-ring PAH: sum of ANT and FLU; OPAH4: sum of BaAQ, BbFLO, BcdPO, and NAPHQ; PAH4: sum of BaA, BaP, BbF, and CHR; vegan: wheat protein based; vegetarian: milk protein based; *n* = 6, each).

To reduce the number of samples and to clearly identify a possible matrix influence, only the four setups for which the highest OPAH and PAH contents were expected were also carried out with both vegan and vegetarian patties (Briq2, Briq2e, Char2, Disp2). For each of these setups, the average contents of OPAH4 and PAH4 were higher in beef patties compared to vegan or vegetarian patties (Fig. 2). This relation was also found for OPAH8 and PAH6 contents (Fig. 3). In contrast to beef patties, the vegan and vegetarian patties had contents below the LOQ for BaAQ, NAPHQ, and BcdPO, the vegetarian patties also for BbFLO and BbF. The lower contents in meat substitute patties can be explained by their lower fat content (Gorji et al., 2016, Lee et al., 2016), which was less than half of the beef patty fat content (beef: 18%, vegan: 7%, vegetarian: 6%). In addition, these patties were barbecued for a shorter time (Briq2 and Char2: 6 min instead of 7 min, Briq2e: 9 min instead of 14 min) since they had already been pre-cooked prior to the sale in contrast to the raw beef patties (Section "Barbecue samples").

To demonstrate extreme conditions that are not to be expected in practical application, the patties for Briq2e were barbecued for a longer time compared to Briq2. All patties from Briq2e exceeded the core temperature of 75 °C and were partially or strongly burnt and therefore no longer suitable for consumption. The median contents of OPAH4 and PAH4 from Briq2e were higher by about 140% and 70% in the beef patties, respectively, as well as 100% and 10% higher in the vegan patties, respectively. However, the increase in medians was significant only for OPAH4 in beef due to the high variances especially with increasing contents (e.g., coefficient of variation (CV) of PAH4 in beef; Briq2: CV = 42%, Briq2e: CV = 66%). For vegetarian patties, the OPAH4 content was below the LOQ in both Briq2 and Briq2e, and the PAH4 contents differed only slightly. Due to the higher contents, differences between setups were principally more evident for beef than for non-meat patties.

The use of lump charcoal (Char2) instead of charcoal briquettes (Briq2) resulted in lower contents of OPAH4 and PAH4 by a median average of about 75% and 40%, respectively, as well as lower OPAH8 and PAH6 contents by about 70% and 50%, respectively, for all patty types. This may be explained by the two different types of heating medium used in these setups. According to the manufacturer, the production of charcoal briquettes uses lump charcoal as well as starch. During barbecuing, it is possible that the starch was incompletely burnt, resulting in higher OPAH and PAH contents. In a study by Kim et al. (2021), beef loin was barbecued in different setups using two types of lump charcoal and charcoal briquettes containing starch. The use of the briquettes with starch resulted in the highest PAH4 content of 96.5 µg/kg.

In all experimental setups using lump charcoal or charcoal briquettes, the total contents of OPAH8 + PAH6 in beef patties were above 20 µg/kg but only for Disp2 in vegan and vegetarian patties (Fig. 3). The low-molecular-weight compounds (3-ring OPAHs and PAHs) accounted for the largest share of the total content of OPAH8 + PAH6 (60–95%) in all setups with lump charcoal or briquettes. Also, the 3-ring OPAHs 9FLO and ATQ were the dominant compounds within OPAHs. The proportion of the toxicologically more relevant compounds OPAH4 and PAH4 of Disp2 (4.2–23.5 µg/kg or 15–30%) was much higher for all three patty-types compared to the other grill types (0.6–3.0 µg/kg or 8–23%).

For the same experimental setups, the relative contents of OPAHs and PAHs differed between patty types (Fig. 3). The vegetarian patties had a higher percentage of 3-ring OPAHs + PAHs (83–95%) and a lower percentage of OPAH4 + PAH4 (2–15%), compared to beef patties with 64–91% 3-ring OPAHs + PAHs, and 8–27% OPAH4 + PAH4. In vegan patties, the proportion of 3-ring OPAHs + PAHs was comparable to vegetarian patties but varied for OPAH4 + PAH4 between experimental setups (see Supplementary Table S3 and S4 for details).

### Relationship between OPAHs, PAHs, and barbecue conditions

It was evaluated if OPAH contents could reliably be estimated from PAH contents. For this purpose, regression models of the entire, unweighted dataset were developed to predict individual or summed OPAHs (Table 2). The focus was on the prediction of the toxicologically more critical group OPAH4 as identified by Clergé et al. (2019). In addition, regressions for all OPAHs combined, i.e. OPAH8, and for three individual OPAHs that could be expected to be correlated to their parent PAHs were analyzed (Table 2; BaPO was not detected in any sample).

**Table 2:** Regressions of individual OPAHs, OPAH4, or OPAH8 on individual or aggregated PAHs or OPAHs (*n* = 108).

**Y**	**X**	**Intercept** **(µg/kg)**	**Coefficient** **(X)**	**Coefficient** **(X^2^)**	**R^2^_adj_**	**RMSE**	**Mean Y** **(µg/kg)**	**RMSE** **(% Mean)**
9FLO	FLU	2.46**	1.99***	−0.05***	0.55	6.13	9.43	65
ATQ	ANT	.	1.66***	−0.12***	0.29	3.31	2.55	130
BaAQ	BaA	−0.04*	0.28***	.	0.74	0.16	0.14	109
BaPO	BaP	.	.	.	.	.	0.00	.
								
OPAH4	ANT	.	1.04***	−0.06*	0.37	2.20	1.74	126
OPAH4	BaA	.	2.49***	0.12*	**0.92**	0.77	1.74	44
OPAH4	BaP	.	3.93***	−0.52***	0.49	1.98	1.74	113
OPAH4	BbF	.	3.57***	−0.15***	**0.97**	0.51	1.74	29
OPAH4	CHR	.	2.97***	.	0.87	1.00	1.74	58
OPAH4	FLU	.	0.33***	.	0.62	1.72	1.74	99
OPAH4	PAH3	.	1.00***	.	**0.94**	0.72	1.74	41
OPAH4	PAH4	.	0.77***	.	0.87	1.01	1.74	58
OPAH4	PAH6	.	0.18***	.	0.69	1.55	1.74	89
								
OPAH4	ATQ	.	0.65***	.	0.80	1.23	1.74	71
OPAH4	**BaAQ**	0.49***	8.75***	.	**0.90**	0.86	1.74	49
OPAH4	**BbFLO**	.	1.45***	.	**0.93**	0.75	1.74	43
OPAH4	**BcdPO**	0.54*	5.57***	−1.03***	0.54	1.89	1.74	108
OPAH4	BZA	−0.18**	2.15***	.	**0.96**	0.58	1.74	33
OPAH4	**NAPHQ**	0.83***	11.23***	.	0.83	1.14	1.74	66
OPAH4	9FLO	.	0.31***	−0.01***	0.21	2.47	1.74	142
								
OPAH8	PAH4	3.91**	6.16***	−0.25***	0.63	8.48	14.61	58
OPAH8	PAH6	2.19*	1.55***	−0.01***	0.81	6.08	14.61	42

OPAH4: BaAQ, BbFLO, BcdPO, and NAPHQ (in bold); PAH3: BaA, BbF, and CHR; PAH4: PAH3 and BaP; PAH6: PAH4, ANT, and FLU.

* P < 0.05, ** P < 0.01, *** P < 0.001.

All models had significant positive linear regression coefficients ranging from as low as 0.18 to a high 11.23, i.e. all individual and summed OPAHs increased with the predictor PAHs or OPAHs. For OPAH4 and PAH3, a perfect linear relation with zero intercept, insignificant quadratic term, and a linear regression coefficient of 1.00 was found (Table 2). Less than half of the models had significant intercepts that were mostly positive. In these cases, individual or summed OPAH contents can on average be expected at some level even if the predictor OPAH or PAH is not detected. In four out of the eight models with intercept, this was as high as 25–31% of the mean Y, and even 48% in the case of OPAH4 and NAPHQ. In the case of three OPAHs that are part of OPAH4, the intercept corresponds to the level of other OPAH4 constituents when the individual predictor OPAH4 is missing (BaAQ, BcdPO and NAPHQ in Table 2). On the other hand, OPAH4 could be detected only from a certain level of BZA, indicated by a negative intercept.

In nearly half of the regression models, also the quadratic term was significant, and in nine out of these ten models, the quadratic regression coefficient was negative. This describes a flattening of the regression with increasing predictor X. Only in one case, OPAH4 increased somewhat exponentially with BaA.

High correlations, with adjusted coefficients of determination R^2^_adj_ ≥ 0.9, were observed for OPAH4 only, in some regressions on PAHs or OPAHs (Table 2). The expected correlation between individual OPAHs and their corresponding parent PAHs was low to moderate with R^2^_adj_ = 0.29–0.74, combined with a high variation (RMSE = 65–130%). In other studies, the correlation coefficient was calculated between 0.89 and 0.98, corresponding to R^2^ = 0.79–0.96 (Gong et al., 2019, Gong et al., 2018b, Hua et al., 2016, Li et al., 2016, Zhao et al., 2017, Zhao et al., 2018). However, in these studies, a separate regression was created for each setup, describing only the correlation within replicates of the same experimental conditions.

The OPAH4 were highly correlated to some PAHs (BaA, BbF, PAH3) and to some OPAHs (BaAQ, BbFLO, BZA) with R^2^_adj_ = 0.90–0.97 (Table 2). Among the predictor OPAHs, only two of the constituent OPAH4 were highly correlated and had RMSE < 50%. This indicates that the processes that affect the levels of constituent OPAH4 during barbecuing differ between these constituents.

The best OPAH predictor for OPAH4 was the non-constituent BZA with R^2^_adj_ = 0.96 and RMSE = 33%. The best overall predictor for OPAH4 was the PAH BbF with R^2^_adj_ = 0.97 and RSME = 29%. The regressions of OPAH4 on the PAH3, PAH4 or PAH6 obtained no higher R^2^_adj_ or lower RMSE than its constituent BbF. Consequently, if OPAH4 is to be predicted from PAH measurements, the best choice would be to use BbF as predictor, which is part of the PAH4 routine analyses. But this is only an option if an RMSE of about 30% is acceptable, which corresponds to a 95% confidence interval of about ± 60% of the mean OPAH4 value. Also, the total content of OPAHs, in this study OPAH8, could not be predicted with high precision: at most, R^2^_adj_ = 0.81 with PAH6 as predictor and RSME = 42%. These results indicate that OPAH and PAH contents are dependent on many different process parameters that need to be further investigated.

**Fig. 4:**
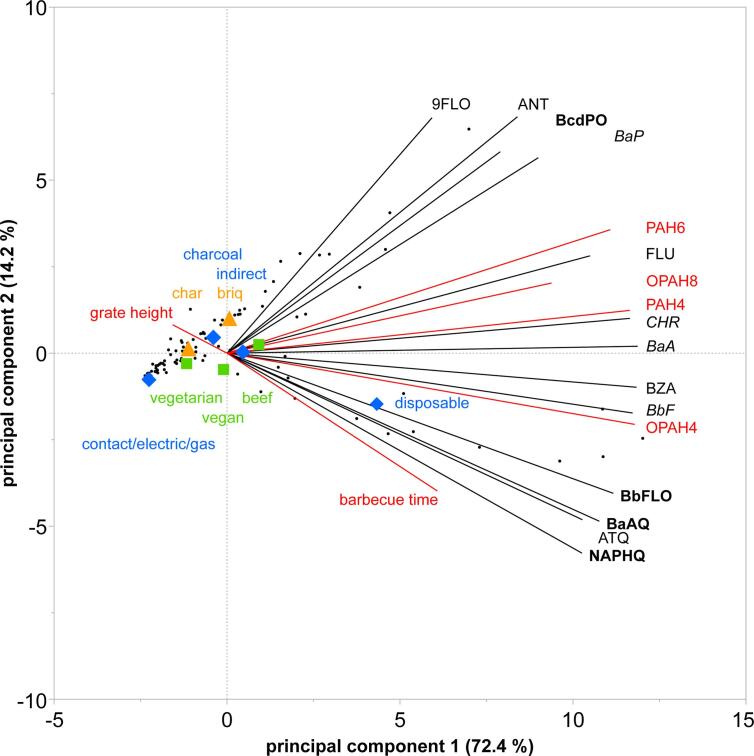
Principal component analysis biplot of the individual OPAH and PAH contents, their summed contents, and the barbecue parameters, specifically barbecue time (in red), grate height (in red), grill type (in blue), heating medium (in yellow), and patty type (in green). (briq: charcoal briquettes; char: lump charcoal; OPAH4: sum of BaAQ, BbFLO, BcdPO, and NAPHQ (in bold); OPAH8: sum of OPAH4, ATQ, BaPO, BZA, and 9FLO; PAH4: sum of BaA, BaP, BbF, and CHR (in italic); PAH6: sum of PAH4, ANT, and FLU; *n* = 108).

For the entire, unweighted dataset, we analyzed the relationship between OPAH and PAH concentrations and the main parameters that defined the barbecue setups, namely grill type, heating medium, grate height, patty type, and barbecue time. The first two components of a PCA explained 87% of the variation in OPAH and PAH concentrations (Fig. 4). The main trend of principal component 1 (PC1) described the overall increase in OPAHs and PAHs, while PC2 added some differentiation between individual OPAHs and PAHs. Three groups emerged in the two-dimensional plot of PC1 and PC2. The main trend of PC1 was closely correlated to a mostly-PAH group 1, composed of the three PAH4 BaA, BbF, and CHR, plus BZA. Less correlated with PC1, and positively with PC2, was a heterogeneous group 2 of two PAHs (ANT, BaP) and two OPAHs (BcdPO, 9FLO). Also, an OPAH group 3 of the three OPAH4 BaAQ, BbFLO and NAPHQ, plus ATQ, was less correlated with PC1, but negatively with PC2. FLU was intermediate between group 1 and group 2, and displayed much the same trend as the two sum contents PAH6 and OPAH8.

The toxicologically relevant subgroups PAH4 and OPAH4 are more closely correlated to PC1, but with PAH4 diverting from the horizontal positively with PC2 (affected by BaP from group 2), and OPAH4 diverting negatively. The latter reflected the 3:1 dichotomy of individual OPAH4 following the trends of group 2 and group 3. BbF correlated most strongly with OPAH4 (Table 2). Within the three groups, no separation was evident between OPAHs and PAHs or by the number of aromatic rings. The pairs of PAHs and their corresponding OPAHs, namely FLU/9FLO, ANT/ATQ and BaA/BaAQ, were less correlated with each other than with other OPAHs or PAHs. In particular, ANT and ATQ nearly had the most diverging directions in the PC1-PC2-plane.

The PCA showed that groups 2 and 3 were at an angle larger than 50° to each other, indicating a different response to variations in barbecue parameters. Group 3, which was mostly composed of the individual OPAH4, was the one with the clearest relationship to barbecue parameters correlated in particular with grill type and barbecue time. The use of a disposable grill instead of a charcoal grill increased the group 3 OPAHs, but had almost no effect on group 2 OPAHs and PAHs. OPAH group 3 was also the one that correlated most with the opposing trends of grate height (decreasing) and barbecue time (increasing), with a larger effect of barbecue time. Compared to the disposable grill, other factors separated less clearly in the overall dataset. Still, a trend could be identified that followed mostly the projection of the heterogeneous group 2. There was a trend of contamination following grill types, in particular charcoal > contact, electric and gas. Within charcoal grills, the heating medium “briquettes” produced higher levels of group 2 OPAHs and PAHs than lump “charcoal”, but heating medium was hardly correlated to the mostly-OPAH4 group 3. The trend of contamination of patty types, namely beef > vegan > vegetarian, was more correlated to group 1 than to group 2 or 3. Overall, the PCA illustrated that individual OPAHs and PAHs more or less differed in their reaction to the effects of parameters in the experimental setups, but showed some common trends in three distinctive groups.

## Conclusions

In this study, the contamination of barbecued products with OPAHs was investigated for the first time. The results showed that different barbecue parameters had a large effect on the OPAH content, especially the choice of grill type and the corresponding heating medium. The highest OPAH as well as PAH contents were generated with a disposable grill, while the lowest contents were generated with electric or gas grills. In the case of barbecuing with a charcoal grill, the OPAH and PAH contents could be reduced by applying a grill with an indirect mode or by increasing the height of the grill grate. Lump charcoal, compared to briquettes, reduced PAH4 contents while OPAH4 contents were at the same level. Also, the type of product to be barbecued had an influence on both OPAH and PAH contents. The non-meat patties had lower levels than the meat patties probably due to their lower fat content. However, all barbecued samples were below the respective maximum levels for BaP and PAH4. But for ATQ, beef patties prepared on the disposable grill exceeded the EU maximum residue level of 10 µg/kg (EU, 2014).

BbF as well as BZA correlated most strongly with OPAH4, but all the regressions showed high variations. No strong correlations of individual OPAHs with the corresponding non-oxygenated PAHs could be detected because the individual OPAHs and PAHs were affected differently by the barbecue conditions. Consequently, we could not find a suitable marker for OPAH4 or OPAH8. Therefore, we recommend to analyze OPAHs individually in addition to PAHs. Furthermore, we expect that OPAH reduction in barbecuing may require additional steps compared to PAH minimization strategies.

## Declaration of Competing Interest

The authors declare that they have no known competing financial interests or personal relationships that could have appeared to influence the work reported in this paper.
