# Supplementary material for: Barbecue conditions affect contents of oxygenated and non-oxygenated polycyclic aromatic hydrocarbons in meat and non-meat patties
Source: Food Chem X. 2022 May 27;14:100351. doi: 10.1016/j.fochx.2022.100351 (PMC9475699; doi:10.1016/j.fochx.2022.100351)
Supplement: Supplementary data 1 [file mmc1.docx]

**Supplementary Material**

Barbecue conditions affect contents of oxygenated and non-oxygenated polycyclic aromatic hydrocarbons in meat and non-meat patties

Lisa Zastrow^a^, Michael Judas^a^, Karl Speer^b^, Karl-Heinz Schwind^a^, Wolfgang Jira^a,*^

^a^ Department of Safety and Quality of Meat, Max Rubner-Institut (MRI), E.-C.-Baumann Straße 20, 95326 Kulmbach, Germany

^b^ Chair of Special Food Chemistry and Food Production, Technical University Dresden, Bergstraße 66, 01069 Dresden, Germany

^*^ Wolfgang.Jira@mri.bund.de, +49 9221/803-313

**Table S1** Ingredients and composition of the commercial patties.

| **Sample material** | **Ingredients ^a^** | **Fat ^b^** | **Carbohydrates ^b^** | **Dietary fiber ^b^** | **Protein ^b^** | **Salt ^b^** |
| --- | --- | --- | --- | --- | --- | --- |
| **Beef patties** | Beef, table salt, spices | 18% | 0.1% | 0% | 20.0% | 1.0% |
| **Vegetarian patties** (milk protein based) | Skim milk, sunflower oil, thickener (calcium alginate, methyl cellulose), chicken egg protein, dextrose, pea protein, wheat gluten, wheat starch, stabilizer (phosphate), acidifier (potassium lactate), oat husk fiber, onion, barley malt extract, tomato, natural flavoring, spices, table salt, iron diphosphate | 5.6% | 6.5% | 5.1% | 15.0% | 2.0% |
| **Vegan patties**  (wheat protein based) | Water, wheat protein, onion, sunflower oil, wheat starch, wheat flour, mustard (water, mustard seed, brandy vinegar, salt, sugar, spices), sugar, thickener (methyl cellulose), salt, spices, hydrolyzed rapeseed protein, dextrose, maltodextrin, garlic, natural flavoring, brandy vinegar, herbs, acidifier (citric acid), barley malt extract | 6.7% | 17.0% | 2.4% | 20% | 1.7% |

**^a^** According to packaging information (descending order).

**^b^** According to packaging information.

**Table S2** Assignment of the OPAHs and PAHs, limit of detection (LOD; in µg/kg), and limit of quantification (LOQ; in µg/kg).

| **Analyte** | **OPAH4 ^a^** | **OPAH8** | **PAH4** | **PAH6** | **Beef patties** | | **Vegetarian patties** ^c^ | | **Vegan patties** ^d^ | |
| --- | --- | --- | --- | --- | --- | --- | --- | --- | --- | --- |
|  |  |  |  |  | **LOD ^b^** | **LOQ ^b^** | **LOD ^b^** | **LOQ ^b^** | **LOD ^b^** | **LOQ ^b^** |
| **ATQ** |  | X |  |  | 0.08 | 0.26 | 0.14 | 0.45 | 0.17 | 0.57 |
| **BaAQ** | X | X |  |  | 0.04 | 0.12 | 0.11 | 0.35 | 0.09 | 0.31 |
| **BaPO** |  | X |  |  | 0.05 | 0.18 | 0.08 | 0.28 | 0.08 | 0.25 |
| **BbFLO** | X | X |  |  | 0.11 | 0.38 | 0.09 | 0.28 | 0.09 | 0.32 |
| **BcdPO** | X | X |  |  | 0.07 | 0.23 | 0.10 | 0.32 | 0.10 | 0.34 |
| **BZA** |  | X |  |  | 0.04 | 0.13 | 0.08 | 0.27 | 0.08 | 0.28 |
| **NAPHQ** | X | X |  |  | 0.06 | 0.19 | 0.08 | 0.27 | 0.09 | 0.30 |
| **9FLO** |  | X |  |  | 0.37 | 1.24 | 0.31 | 1.03 | 0.43 | 1.43 |
| **ANT** |  |  |  | X | 0.03 | 0.10 | 0.06 | 0.21 | 0.07 | 0.24 |
| **BaA** |  |  | X | X | 0.03 | 0.11 | 0.08 | 0.25 | 0.07 | 0.25 |
| **BaP** |  |  | X | X | 0.04 | 0.13 | 0.09 | 0.28 | 0.08 | 0.25 |
| **BbF** |  |  | X | X | 0.06 | 0.19 | 0.11 | 0.37 | 0.06 | 0.19 |
| **CHR** |  |  | X | X | 0.04 | 0.13 | 0.10 | 0.33 | 0.07 | 0.25 |
| **FLU** |  |  |  | X | 0.06 | 0.19 | 0.08 | 0.28 | 0.17 | 0.55 |

^a^ Toxicological most relevant OPAHs according to Clerge et al. (2019).

^b^ For method validation, see Zastrow et al. (2021).

^c^ Milk protein based.

^d^ Wheat protein based.

**Table S3** Median contents of OPAHs in barbecued beef, vegan, and vegetarian patties (in µg/kg; *n* = 6, each).

| **Patty type** | **Setup** | **ATQ** | **BaAQ** | **BaPO** | **BbFLO** | **BcdPO** | **BZA** | **NAPHQ** | **9FLO** | **OPAH4** | **OPAH8** |
| --- | --- | --- | --- | --- | --- | --- | --- | --- | --- | --- | --- |
| **Beef** | Briq2 | 1.9 | 0.1 | *< LOD^c^* | 1.3 | 0.2 | 1.3 | *< LOQ^c^* | 24.9 | 1.6 | 29.6 |
|  | Briq4 | 1.6 | 0.1 | *< LOD^c^* | 1.0 | 1.1 | 1.0 | *< LOQ^c^* | 14.7 | 2.2 | 19.4 |
|  | Briq8 | 1.1 | *< LOQ^c^* | *< LOD^c^* | 0.5 | *< LOQ^c^* | 0.5 | *< LOD^c^* | 11.7 | 0.5 | 13.8 |
|  | Briq2e | 2.1 | 0.2 | *< LOD^c^* | 1.8 | 1.3 | 2.4 | 0.1 | 24.4 | 3.5 | 32.3 |
|  | Disp2 | 15.7 | 1.2 | *< LOD^c^* | 8.0 | 1.0 | 5.0 | 0.9 | 12.9 | 11.1 | 44.7 |
|  | Char2 | 2.3 | *< LOQ^c^* | *< LOD^c^* | 1.3 | *< LOQ^c^* | 0.8 | *< LOQ^c^* | 15.8 | 1.3 | 20.2 |
|  | Ind | 0.4 | *< LOQ^c^* | *< LOD^c^* | *< LOQ^c^* | *< LOD^c^* | *< LOQ^c^* | *< LOD^c^* | *< LOQ^c^* | - | 0.4 |
|  | Cont0 | 0.3 | *< LOD^c^* | *< LOD^c^* | *< LOD^c^* | *< LOD^c^* | *< LOD^c^* | *< LOD^c^* | *< LOD^c^* | - | 0.3 |
|  | Elec4 | 0.3 | *< LOD^c^* | *< LOD^c^* | *< LOD^c^* | *< LOD^c^* | *< LOD^c^* | *< LOD^c^* | *< LOQ^c^* | - | 0.3 |
|  | Gas7 | 0.3 | *< LOD^c^* | *< LOD^c^* | *< LOD^c^* | *< LOD^c^* | *< LOD^c^* | *< LOD^c^* | *< LOQ^c^* | - | 0.3 |
| **Vegan ^a^** | Briq2 | 0.9 | *< LOQ^c^* | *< LOD^c^* | 0.6 | *< LOQ^c^* | 0.5 | *< LOQ^c^* | 6.0 | 0.6 | 8.0 |
|  | Briq2e | 1.2 | *< LOQ^c^* | *< LOD^c^* | 0.6 | 0.5 | 0.7 | *< LOQ^c^* | 4.6 | 1.0 | 7.4 |
|  | Disp2 | 6.3 | 0.6 | *< LOD^c^* | 3.6 | 0.5 | 2.1 | 0.4 | 5.2 | 5.2 | 18.7 |
|  | Char2 | 1.0 | *< LOQ^c^* | *< LOD^c^* | 0.5 | *< LOD^c^* | 0.3 | *< LOD^c^* | 7.1 | 0.5 | 8.8 |
| **Vegetarian ^b^** | Briq2 | 0.8 | *< LOQ^c^* | *< LOD^c^* | *< LOQ^c^* | *< LOQ^c^* | 0.2 | *< LOD^c^* | 7.0 | - | 8.0 |
|  | Briq2e | 1.0 | *< LOQ^c^* | *< LOD^c^* | *< LOQ^c^* | *< LOQ^c^* | 0.3 | *< LOD^c^* | 4.5 | - | 5.9 |
|  | Disp2 | 5.0 | 0.2 | *< LOD^c^* | 1.6 | 0.2 | 0.7 | *< LOQ^c^* | 11.5 | 2.1 | 19.3 |
|  | Char2 | 0.8 | *< LOQ^c^* | *< LOD^c^* | *< LOQ^c^* | *< LOD^c^* | *< LOQ^c^* | *< LOD^c^* | 2.3 | - | 3.1 |

^a^ Wheat protein based.

^b^ Milk protein based.

^c^ LOD and LOQ are shown in Table S2.

**Table S4** Median contents of PAHs in barbecued beef, vegan, and vegetarian patties (in µg/kg; *n* = 6, each).

| **Patty type** | **Setup** | **ANT** | **BaA** | **BaP** | **BbF** | **CHR** | **FLU** | **PAH4** | **PAH6** |
| --- | --- | --- | --- | --- | --- | --- | --- | --- | --- |
| **Beef** | Briq2 | 7.9 | 0.9 | 1.0 | 0.6 | 0.8 | 12.1 | 3.3 | 23.3 |
|  | Briq4 | 5.1 | 0.8 | 1.0 | 0.7 | 0.8 | 9.9 | 3.2 | 18.2 |
|  | Briq8 | 2.7 | 0.4 | 0.2 | 0.3 | 0.4 | 6.0 | 1.2 | 10.0 |
|  | Briq2e | 5.7 | 1.5 | 1.7 | 1.2 | 1.3 | 9.7 | 5.7 | 21.0 |
|  | Disp2 | 5.0 | 3.4 | 1.7 | 3.5 | 2.9 | 22.0 | 11.6 | 38.6 |
|  | Char2 | 5.3 | 0.5 | 0.2 | 0.4 | 0.4 | 10.6 | 1.6 | 17.5 |
|  | Ind | 0.2 | 0.2 | *< LOQ^c^* | *< LOQ^c^* | 0.2 | 0.3 | 0.4 | 0.9 |
|  | Cont0 | *< LOD^c^* | *< LOQ^c^* | *< LOQ^c^* | *< LOD^c^* | *< LOD^c^* | *< LOD^c^* | - | - |
|  | Elec4 | *< LOD^c^* | *< LOQ^c^* | *< LOQ^c^* | *< LOQ^c^* | *< LOD^c^* | *< LOQ^c^* | - | - |
|  | Gas7 | *< LOD^c^* | *< LOQ^c^* | *< LOQ^c^* | *< LOQ^c^* | *< LOD^c^* | *< LOQ^c^* | - | - |
| **Vegan ^a^** | Briq2 | 0.8 | 0.3 | 0.7 | 0.3 | 0.4 | 2.2 | 1.6 | 4.6 |
|  | Briq2e | 1.1 | 0.3 | 0.8 | 0.4 | 0.3 | 2.6 | 1.8 | 3.2 |
|  | Disp2 | 2.6 | 1.6 | 0.8 | 1.5 | 1.6 | 7.4 | 5.6 | 15.6 |
|  | Char2 | 0.6 | 0.2 | 0.1 | 0.1 | 0.1 | 1.5 | 0.5 | 2.6 |
| **Vegetarian ^b^** | Briq2 | 0.5 | 0.1 | 0.6 | *< LOQ^c^* | 0.2 | 0.8 | 0.9 | 2.2 |
|  | Briq2e | 0.4 | 0.1 | 0.3 | *< LOQ^c^* | 0.2 | 0.9 | 0.6 | 1.9 |
|  | Disp2 | 4.7 | 0.7 | 0.3 | 0.5 | 0.6 | 2.8 | 1.9 | 9.4 |
|  | Char2 | *< LOQ^c^* | *< LOQ^c^* | *< LOQ^c^* | *< LOQ^c^* | *< LOQ^c^* | 0.4 | - | 0.4 |

^a^ Wheat protein based.

^b^ Milk protein based.

^c^ LOD and LOQ are shown in Table S2.

**Table S5** Mean contents and standard deviations of OPAHs in barbecued beef, vegan, and vegetarian patties (in µg/kg; *n* = 6, each).

| **Patty type** | **Setup** | **ATQ** | **BaAQ** | **BaPO** | **BbFLO** | **BcdPO** | **BZA** | **NAPHQ** | **9FLO** | **OPAH4** | **OPAH8** |
| --- | --- | --- | --- | --- | --- | --- | --- | --- | --- | --- | --- |
| **Beef** | Briq2 | 2.2 ± 0.7 | 0.1 ± 0.1* | *< LOD^c^* | 1.4 ± 0.3 | 0.4 ± 0.7* | 1.3 ± 0.4 | *< LOQ^c^* | 27.2 ± 9.8 | 1.9 ± 1.1 | 32.6 ± 12.0* |
|  | Briq4 | 1.6 ± 0.3 | 0.1 ± 0.1 | *< LOD^c^* | 1.0 ± 0.3 | 1.6 ± 1.9 | 1.3 ± 0.8 | *< LOQ^c^* | 16.1 ± 2.8 | 2.7 ± 2.3 | 21.7 ± 6.2 |
|  | Briq8 | 1.2 ± 0.4 | *< LOQ^c^* | *< LOD^c^* | 0.6 ± 0.2 | *< LOQ^c^* | 0.4 ± 0.1 | *< LOD^c^* | 12.5 ± 2.8 | 0.6 ± 0.2 | 14.7 ± 3.5 |
|  | Briq2e | 2.4 ± 0.5* | 0.2 ± 0.1 | *< LOD^c^* | 1.8 ± 0.4 | 1.7 ± 1.6 | 2.2 ± 0.6 | 0.1 ± 0.2* | 25.7 ± 5.1 | 3.8 ± 2.3 | 34.1 ± 8.5 |
|  | Disp2 | 16.2 ± 2.9 | 1.2 ± 0.2 | *< LOD^c^* | 7.6 ± 1.3 | 1.2 ± 0.7 | 5.0 ± 1.2 | 0.9 ± 0.2 | 14.4 ± 3.5 | 10.9 ± 2.4 | 46.5 ± 10.0 |
|  | Char2 | 2.7 ± 1.1 | *< LOQ^c^* | *< LOD^c^* | 1.4 ± 0.7* | *< LOQ^c^* | 1.0 ± 0.5* | *< LOQ^c^* | 16.2 ± 2.6 | 1.4 ± 0.7* | 21.3 ± 5.9 |
|  | Ind | 0.4 ± 0.1 | *< LOQ^c^* | *< LOD^c^* | *< LOQ^c^* | *< LOD^c^* | *< LOQ^c^* | *< LOD^c^* | *< LOQ^c^* | - | 0.4 ± 0.1 |
|  | Cont0 | 0.2 ± 0.1* | *< LOD^c^* | *< LOD^c^* | *< LOD^c^* | *< LOD^c^* | *< LOD^c^* | *< LOD^c^* | *< LOD^c^* | - | 0.2 ± 0.1* |
|  | Elec4 | 0.3 ± 0.1* | *< LOD^c^* | *< LOD^c^* | *< LOD^c^* | *< LOD^c^* | *< LOD^c^* | *< LOD^c^* | *< LOQ^c^* | - | 0.3 ± 0.1* |
|  | Gas7 | 0.4 ± 0.1 | *< LOD^c^* | *< LOD^c^* | *< LOD^c^* | *< LOD^c^* | *< LOD^c^* | *< LOD^c^* | *< LOQ^c^* | - | 0.4 ± 0.1 |
| **Vegan ^a^** | Briq2 | 0.9 ± 0.1 | *< LOQ^c^* | *< LOD^c^* | 0.5 ± 0.3* | *< LOQ^c^* | 0.5 ± 0.1 | *< LOQ^c^* | 5.7 ± 1.3 | 0.5 ± 0.3* | 7.6 ± 1.8 |
|  | Briq2e | 1.2 ± 0.2* | *< LOQ^c^* | *< LOD^c^* | 0.6 ± 0.2 | 0.6 ± 0.7 | 0.7 ± 0.2 | *< LOQ^c^* | 5.0 ± 2.1 | 1.2 ± 0.9 | 8.1 ± 3.4* |
|  | Disp2 | 6.1 ± 2.0 | 0.6 ± 0.2 | *< LOD^c^* | 3.6 ± 1.2 | 0.6 ± 0.1 | 2.1 ± 0.8 | 0.4 ± 0.2 | 6.0 ± 3.1 | 5.2 ± 1.7 | 19.4 ± 7.6 |
|  | Char2 | 0.9 ± 0.3 | *< LOQ^c^* | *< LOD^c^* | 0.4 ± 0.3 | *< LOD^c^* | 0.3 ± 0.2 | *< LOD^c^* | 9.8 ± 5.7 | 0.4 ± 0.3 | 11.4 ± 6.5 |
| **Vegetarian ^b^** | Briq2 | 0.8 ± 0.2 | *< LOQ^c^* | *< LOD^c^* | *< LOQ^c^* | *< LOQ^c^* | 0.2 ± 0.1 | *< LOD^c^* | 12.4 ± 11.8* | - | 13.4 ± 12.1* |
|  | Briq2e | 1.1 ± 0.3 | *< LOQ^c^* | *< LOD^c^* | *< LOQ^c^* | *< LOQ^c^* | 0.3 ± 0.1 | *< LOD^c^* | 5.1 ± 2.4 | - | 6.5 ± 2.8 |
|  | Disp2 | 6.6 ± 3.6 | 0.2 ± 0.1 | *< LOD^c^* | 1.7 ± 0.7 | 0.2 ± 0.1 | 0.7 ± 0.3 | *< LOQ^c^* | 11.2 ± 4.2 | 2.1 ± 0.9 | 20.6 ± 9.0 |
|  | Char2 | 0.8 ± 0.2 | *< LOQ^c^* | *< LOD^c^* | *< LOQ^c^* | *< LOD^c^* | *< LOQ^c^* | *< LOD^c^* | 2.4 ± 0.9 | - | 3.2 ± 1.1 |

^a^ Wheat protein based.

^b^ Milk protein based.

^c^ LOD and LOQ are shown in Table S2.

* Significant deviation from normal distribution (P < 0.05).

**Table S6** Mean contents and standard deviations of PAHs in barbecued beef, vegan, and vegetarian patties (in µg/kg; *n* = 6, each).

| **Patty type** | **Setup** | **ANT** | **BaA** | **BaP** | **BbF** | **CHR** | **FLU** | **PAH4** | **PAH6** |
| --- | --- | --- | --- | --- | --- | --- | --- | --- | --- |
| **Beef** | Briq2 | 7.7 ± 2.0 | 1.0 ± 0.3 | 1.1 ± 0.6 | 0.6 ± 0.2* | 0.9 ± 0.3* | 12.5 ± 3.8 | 3.6 ± 1.4* | 23.8 ± 7.2 |
|  | Briq4 | 5.0 ± 1.3 | 1.1 ± 0.7 | 1.3 ± 1.2 | 0.9 ± 0.6 | 1.1 ± 0.8 | 10.7 ± 2.8 | 4.4 ± 3.3 | 20.1 ± 7.4 |
|  | Briq8 | 2.8 ± 1.0 | 0.4 ± 0.1 | 0.3 ± 0.1 | 0.2 ± 0.2 | 0.3 ± 0.1 | 6.5 ± 2.7 | 1.2 ± 0.5 | 10.5 ± 4.2 |
|  | Briq2e | 7.5 ± 3.5* | 1.7 ± 1.0 | 2.0 ± 1.7 | 1.2 ± 0.6 | 1.7 ± 1.0 | 12.3 ± 6.5* | 6.6 ± 4.3 | 26.4 ± 14.3* |
|  | Disp2 | 5.3 ± 1.3 | 3.3 ± 0.8 | 1.7 ± 0.9 | 3.4 ± 1.0 | 2.9 ± 0.8 | 21.4 ± 9.3 | 11.3 ± 3.5 | 38.0 ± 14.1 |
|  | Char2 | 5.4 ± 1.6 | 0.6 ± 0.2* | 0.3 ± 0.1* | 0.4 ± 0.2* | 0.5 ± 0.2* | 12.0 ± 4.5 | 1.8 ± 0.7* | 19.2 ± 6.8 |
|  | Ind | 0.3 ± 0.3 | 0.2 ± 0.2 | *< LOQ^c^* | *< LOQ^c^* | 0.2 ± 0.1 | 0.3 ± 0.3 | 0.4 ± 0.3 | 1.0 ± 0.9 |
|  | Cont0 | *< LOD^c^* | *< LOQ^c^* | *< LOQ^c^* | *< LOD^c^* | *< LOD^c^* | *< LOD^c^* | - | - |
|  | Elec4 | *< LOD^c^* | *< LOQ^c^* | *< LOQ^c^* | *< LOQ^c^* | *< LOD^c^* | *< LOQ^c^* | - | - |
|  | Gas7 | *< LOD^c^* | *< LOQ^c^* | *< LOQ^c^* | *< LOQ^c^* | *< LOD^c^* | *< LOQ^c^* | - | - |
| **Vegan ^a^** | Briq2 | 0.8 ± 0.2 | 0.3 ± 0.1 | 0.7 ± 0.4 | 0.3 ± 0.1 | 0.4 ± 0.1 | 1.9 ± 0.6 | 1.7 ± 0.7 | 4.4 ± 1.5* |
|  | Briq2e | 1.0 ± 0.4 | 0.4 ± 0.3 | 1.0 ± 0.6 | 0.5 ± 0.2 | 0.4 ± 0.2* | 2.5 ± 0.4 | 2.3 ± 1.3* | 5.8 ± 2.1 |
|  | Disp2 | 2.9 ± 1.0 | 1.7 ± 0.7 | 0.9 ± 0.3 | 1.6 ± 0.6 | 1.6 ± 0.7 | 7.1 ± 1.5 | 5.8 ± 2.3 | 15.8 ± 4.8 |
|  | Char2 | 0.5 ± 0.4 | 0.1 ± 0.1 | 0.1 ± 0.1 | 0.1 ± 0.1* | 0.1 ± 0.1* | 1.5 ± 0.6 | 0.4 ± 0.4 | 2.4 ± 1.4 |
| **Vegetarian ^b^** | Briq2 | 0.6 ± 0.4 | 0.1 ± 0.1 | 0.6 ± 0.4 | *< LOQ^c^* | 0.2 ± 0.1 | 0.9 ± 0.3* | 0.9 ± 0.6 | 2.4 ± 1.3 |
|  | Briq2e | 0.6 ± 0.5* | 0.2 ± 0.3* | 0.4 ± 0.2 | *< LOQ^c^* | 0.3 ± 0.2* | 1.0 ± 0.4 | 0.9 ± 0.7 | 2.5 ± 1.6 |
|  | Disp2 | 4.5 ± 1.3 | 0.8 ± 0.4 | 0.3 ± 0.1 | 0.5 ± 0.2 | 0.6 ± 0.3 | 3.8 ± 3.3 | 2.2 ± 1.0 | 10.5 ± 5.6 |
|  | Char2 | *< LOQ^c^* | *< LOQ^c^* | *< LOQ^c^* | *< LOQ^c^* | *< LOQ^c^* | 0.6 ± 0.6 | - | 0.6 ± 0.6 |

^a^ Wheat protein based.

^b^ Milk protein based.

^c^ LOD and LOQ are shown in Table S2.

* Significant deviation from normal distribution (P < 0.05).


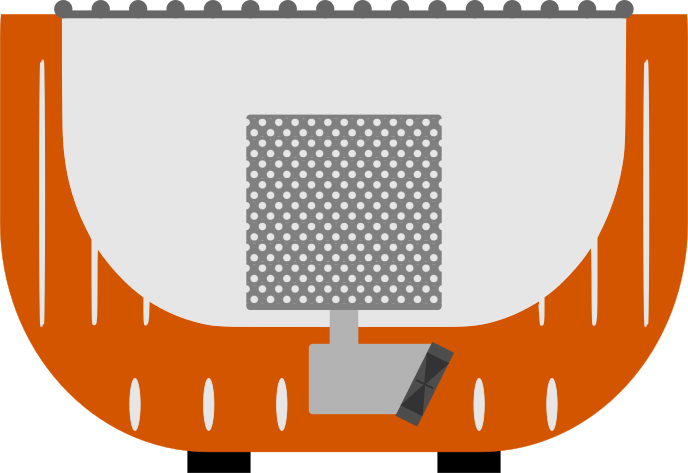

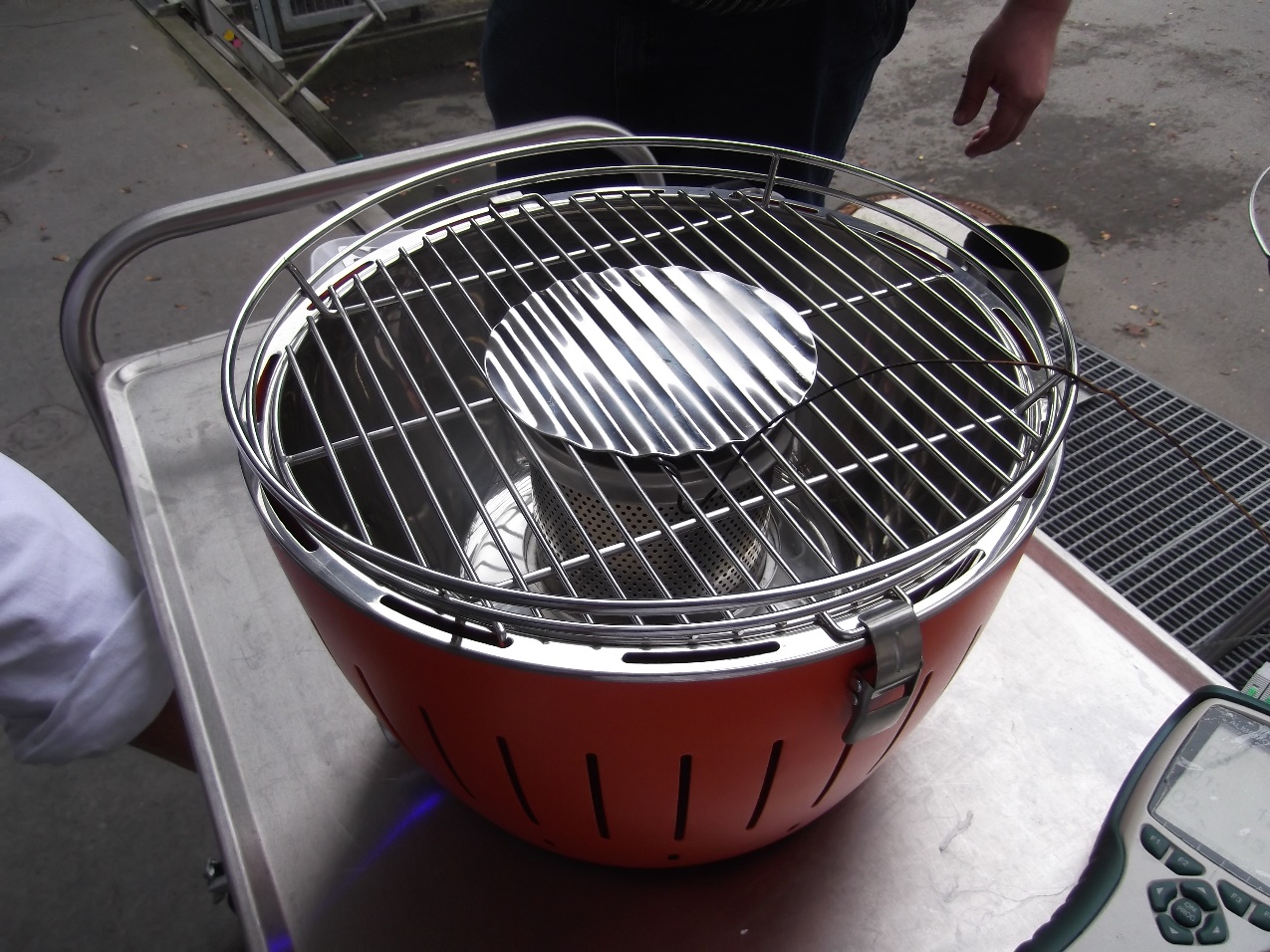


fire box with lid

fan

grill grate

**Figure S1** Construction of the indirect grill of setup “Ind”.


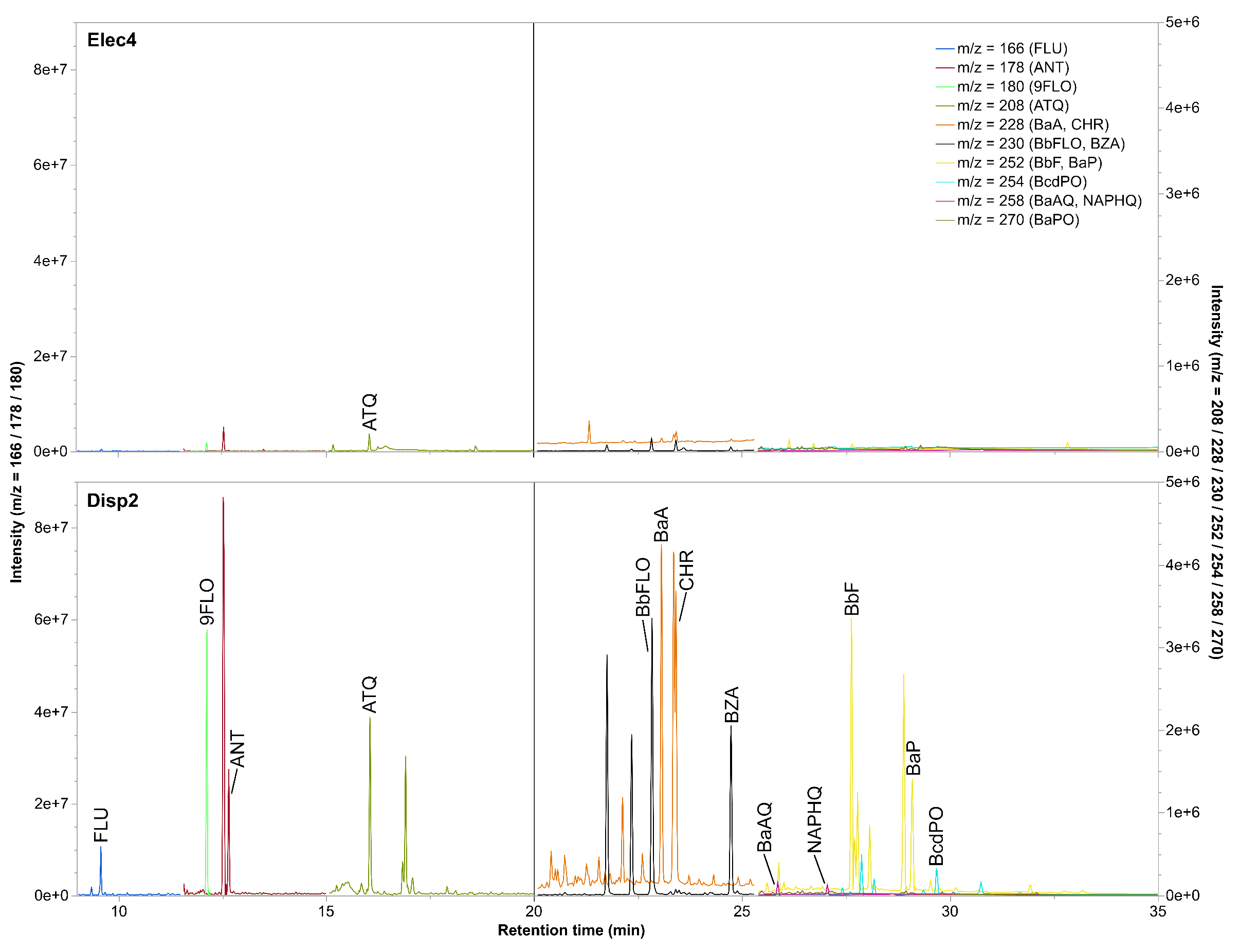


**Figure S2** Exemplary chromatograms of the beef patties with low (Elec4) or high (Disp2) OPAH and PAH contamination. The solid vertical line shows the switch to the right y-axis. The listed m/z ratios are rounded. Baseline separations were obtained for the isomers ANT/phenanthrene, BbF/benzo[j]fluoranthene/benzo[k]fluoranthene, and BaP/benzo[e]pyrene. CHR and triphenylene were sufficiently separated to determine the isolated CHR content.
